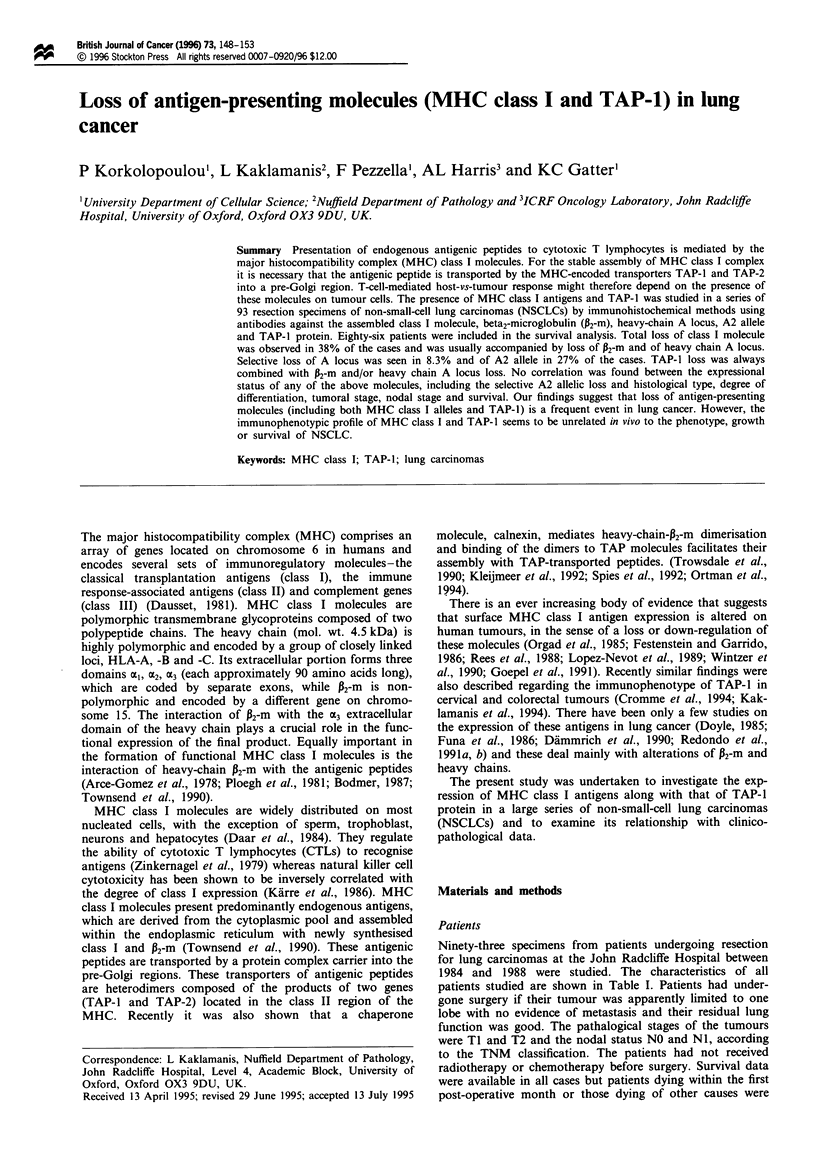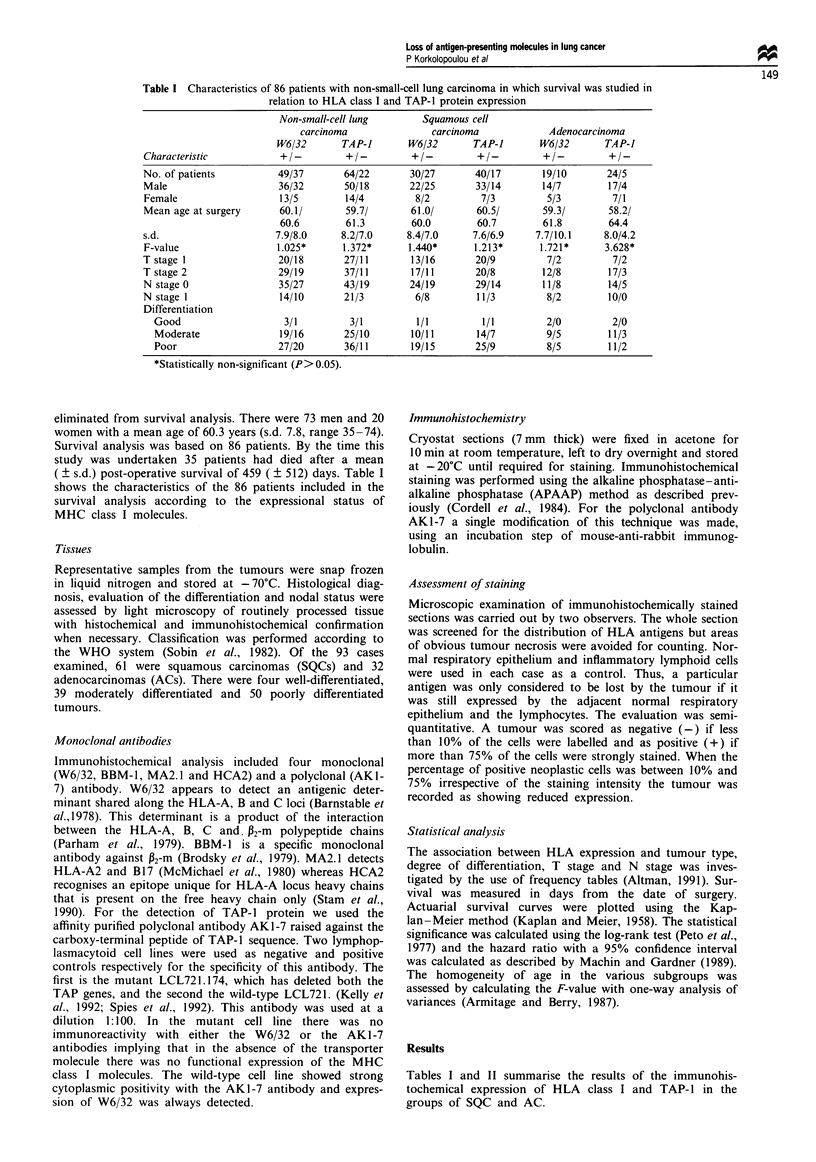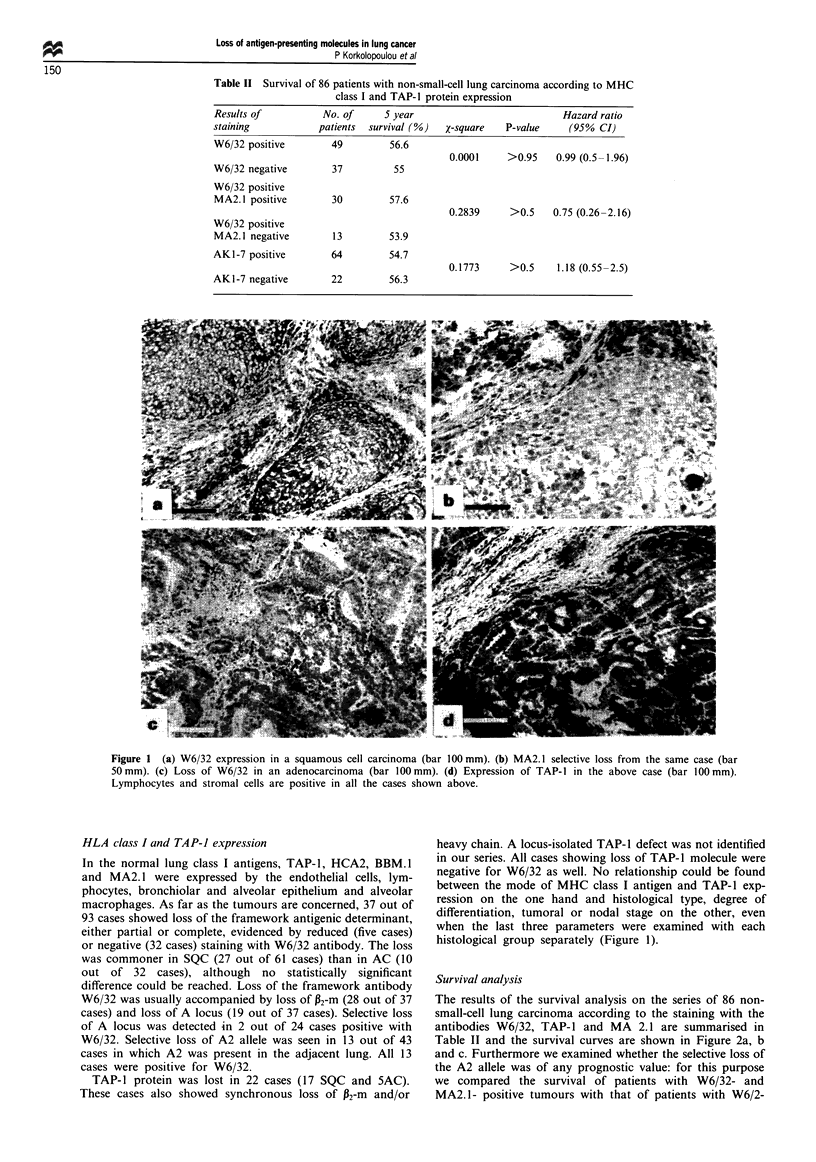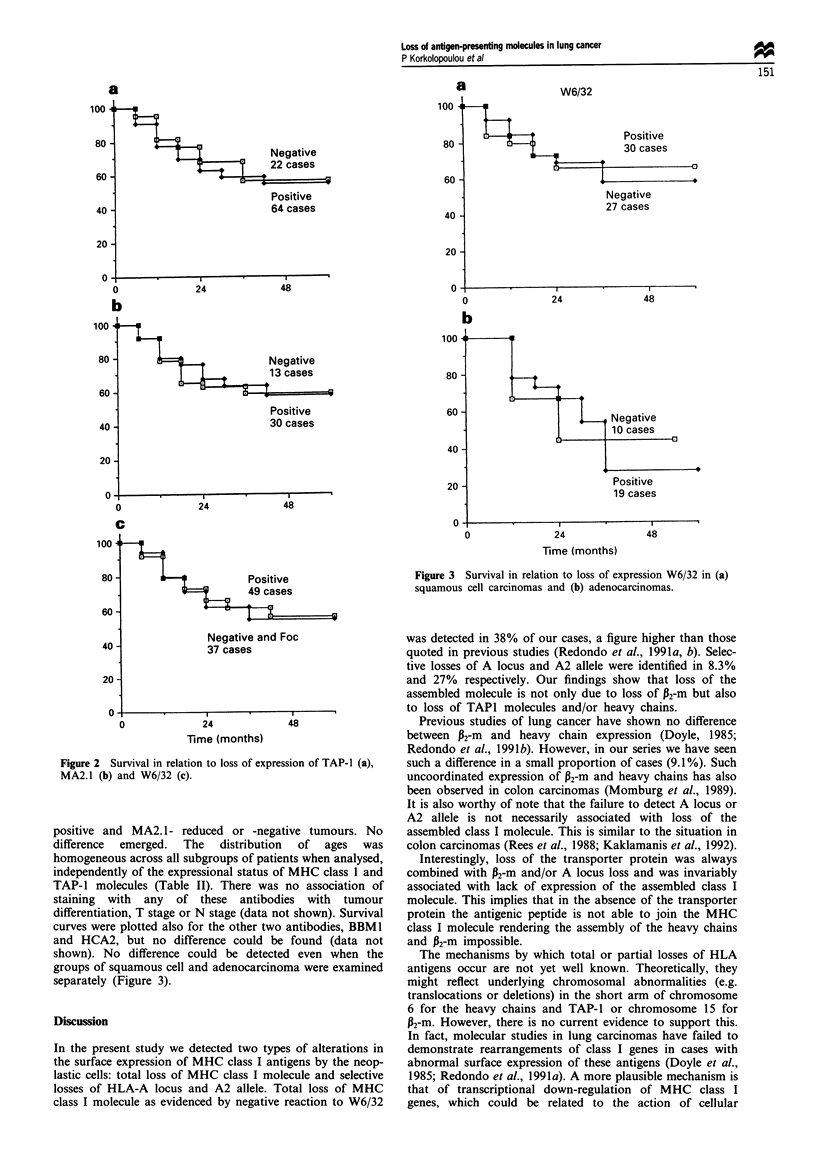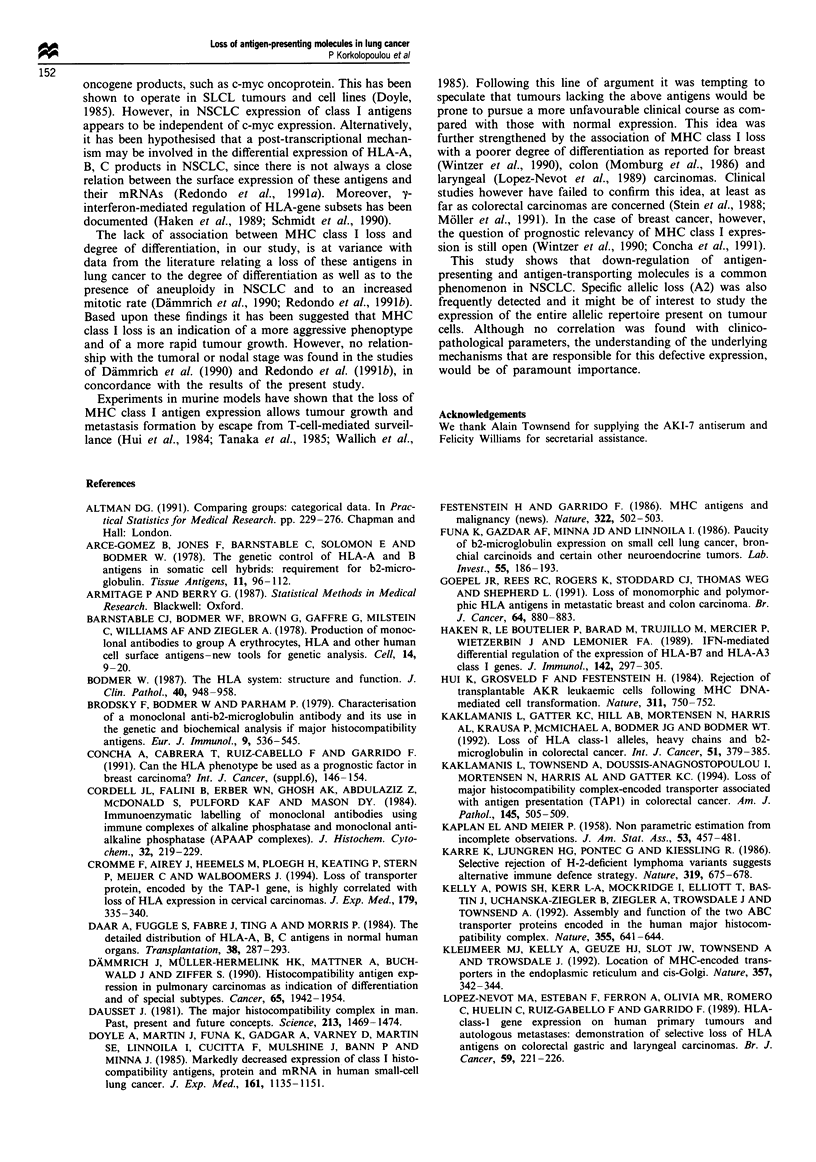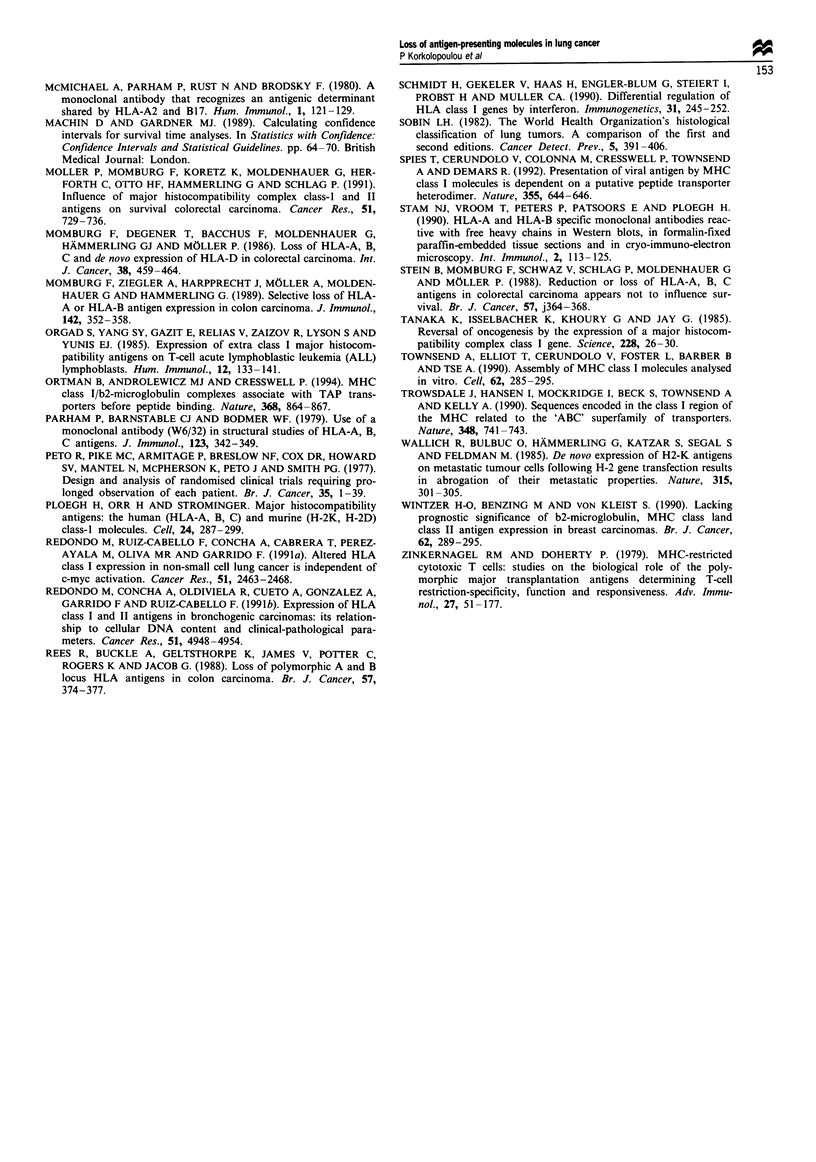# Loss of antigen-presenting molecules (MHC class I and TAP-1) in lung cancer.

**DOI:** 10.1038/bjc.1996.28

**Published:** 1996-01

**Authors:** P. Korkolopoulou, L. Kaklamanis, F. Pezzella, A. L. Harris, K. C. Gatter

**Affiliations:** University Department of Cellular Science, John Radcliffe Hospital, University of Oxford, UK.

## Abstract

**Images:**


British Journal of Cancer (1996) 73, 148-153
© 1996 Stockton Press All rights reserved 0007-0920/96 $12.00

### Loss of antigen-presenting molecules (MHC class I and TAP-1) in lung cancer

P Korkolopoulou[1], L Kaklamanis[2], F Pezzella[1], AL Harris[3] and KC Gatter[1]

[1]*University Department of Cellular Science;* [2]*Nuffield Department of Pathology and* [3]*ICRF Oncology Laboratory, John Radcliffe Hospital, University of Oxford, Oxford OX3 9DU, UK.*

**Summary** Presentation of endogenous antigenic peptides to cytotoxic T lymphocytes is mediated by the major histocompatibility complex (MHC) class I molecules. For the stable assembly of MHC class I complex it is necessary that the antigenic peptide is transported by the MHC-encoded transporters TAP-1 and TAP-2 into a pre-Golgi region. T-cell-mediated host-*vs*-tumour response might therefore depend on the presence of these molecules on tumour cells. The presence of MHC class I antigens and TAP-1 was studied in a series of 93 resection specimens of non-small-cell lung carcinomas (NSCLCs) by immunohistochemical methods using antibodies against the assembled class I molecule, beta$_2$-microglobulin ($\beta_2$-m), heavy-chain A locus, A2 allele and TAP-1 protein. Eighty-six patients were included in the survival analysis. Total loss of class I molecule was observed in 38% of the cases and was usually accompanied by loss of $\beta_2$-m and of heavy chain A locus. Selective loss of A locus was seen in 8.3% and of A2 allele in 27% of the cases. TAP-1 loss was always combined with $\beta_2$-m and/or heavy chain A locus loss. No correlation was found between the expressional status of any of the above molecules, including the selective A2 allelic loss and histological type, degree of differentiation, tumoral stage, nodal stage and survival. Our findings suggest that loss of antigen-presenting molecules (including both MHC class I alleles and TAP-1) is a frequent event in lung cancer. However, the immunophenotypic profile of MHC class I and TAP-1 seems to be unrelated *in vivo* to the phenotype, growth or survival of NSCLC.

**Keywords:** MHC class I; TAP-1; lung carcinomas

The major histocompatibility complex (MHC) comprises an array of genes located on chromosome 6 in humans and encodes several sets of immunoregulatory molecules–the classical transplantation antigens (class I), the immune response-associated antigens (class II) and complement genes (class III) (Dausset, 1981). MHC class I molecules are polymorphic transmembrane glycoproteins composed of two polypeptide chains. The heavy chain (mol. wt. 4.5 kDa) is highly polymorphic and encoded by a group of closely linked loci, HLA-A, -B and -C. Its extracellular portion forms three domains $\alpha_1$, $\alpha_2$, $\alpha_3$ (each approximately 90 amino acids long), which are coded by separate exons, while $\beta_2$-m is non-polymorphic and encoded by a different gene on chromosome 15. The interaction of $\beta_2$-m with the $\alpha_3$ extracellular domain of the heavy chain plays a crucial role in the functional expression of the final product. Equally important in the formation of functional MHC class I molecules is the interaction of heavy-chain $\beta_2$-m with the antigenic peptides (Arce-Gomez *et al.*, 1978; Ploegh *et al.*, 1981; Bodmer, 1987; Townsend *et al.*, 1990).

MHC class I molecules are widely distributed on most nucleated cells, with the exception of sperm, trophoblast, neurons and hepatocytes (Daar *et al.*, 1984). They regulate the ability of cytotoxic T lymphocytes (CTLs) to recognise antigens (Zinkernagel *et al.*, 1979) whereas natural killer cell cytotoxicity has been shown to be inversely correlated with the degree of class I expression (Kärre *et al.*, 1986). MHC class I molecules present predominantly endogenous antigens, which are derived from the cytoplasmic pool and assembled within the endoplasmic reticulum with newly synthesised class I and $\beta_2$-m (Townsend *et al.*, 1990). These antigenic peptides are transported by a protein complex carrier into the pre-Golgi regions. These transporters of antigenic peptides are heterodimers composed of the products of two genes (TAP-1 and TAP-2) located in the class II region of the MHC. Recently it was also shown that a chaperone molecule, calnexin, mediates heavy-chain-$\beta_2$-m dimerisation and binding of the dimers to TAP molecules facilitates their assembly with TAP-transported peptides. (Trowsdale *et al.*, 1990; Kleijmeer *et al.*, 1992; Spies *et al.*, 1992; Ortman *et al.*, 1994).

There is an ever increasing body of evidence that suggests that surface MHC class I antigen expression is altered on human tumours, in the sense of a loss or down-regulation of these molecules (Orgad *et al.*, 1985; Festenstein and Garrido, 1986; Rees *et al.*, 1988; Lopez-Nevot *et al.*, 1989; Wintzer *et al.*, 1990; Goepel *et al.*, 1991). Recently similar findings were also described regarding the immunophenotype of TAP-1 in cervical and colorectal tumours (Cromme *et al.*, 1994; Kaklamanis *et al.*, 1994). There have been only a few studies on the expression of these antigens in lung cancer (Doyle, 1985; Funa *et al.*, 1986; Dämmrich *et al.*, 1990; Redondo *et al.*, 1991*a*, *b*) and these deal mainly with alterations of $\beta_2$-m and heavy chains.

The present study was undertaken to investigate the expression of MHC class I antigens along with that of TAP-1 protein in a large series of non-small-cell lung carcinomas (NSCLCs) and to examine its relationship with clinicopathological data.

### Materials and methods

### *Patients*

Ninety-three specimens from patients undergoing resection for lung carcinomas at the John Radcliffe Hospital between 1984 and 1988 were studied. The characteristics of all patients studied are shown in Table I. Patients had undergone surgery if their tumour was apparently limited to one lobe with no evidence of metastasis and their residual lung function was good. The pathalogical stages of the tumours were T1 and T2 and the nodal status N0 and N1, according to the TNM classification. The patients had not received radiotherapy or chemotherapy before surgery. Survival data were available in all cases but patients dying within the first post-operative month or those dying of other causes were

Correspondence: L Kaklamanis, Nuffield Department of Pathology, John Radcliffe Hospital, Level 4, Academic Block, University of Oxford, Oxford OX3 9DU, UK.
Received 13 April 1995; revised 29 June 1995; accepted 13 July 1995

**Table I** Characteristics of 86 patients with non-small-cell lung carcinoma in which survival was studied in relation to HLA class I and TAP-1 protein expression

| | *Non-small-cell lung carcinoma* | | *Squamous cell carcinoma* | | *Adenocarcinoma* | |
|---|---|---|---|---|---|---|
| *Characteristic* | *W6/32* +/− | *TAP-1* +/− | *W6/32* +/− | *TAP-1* +/− | *W6/32* +/− | *TAP-1* +/− |
| No. of patients | 49/37 | 64/22 | 30/27 | 40/17 | 19/10 | 24/5 |
| Male | 36/32 | 50/18 | 22/25 | 33/14 | 14/7 | 17/4 |
| Female | 13/5 | 14/4 | 8/2 | 7/3 | 5/3 | 7/1 |
| Mean age at surgery | 60.1/ 60.6 | 59.7/ 61.3 | 61.0/ 60.0 | 60.5/ 60.7 | 59.3/ 61.8 | 58.2/ 64.4 |
| s.d. | 7.9/8.0 | 8.2/7.0 | 8.4/7.0 | 7.6/6.9 | 7.7/10.1 | 8.0/4.2 |
| F-value | 1.025* | 1.372* | 1.440* | 1.213* | 1.721* | 3.628* |
| T stage 1 | 20/18 | 27/11 | 13/16 | 20/9 | 7/2 | 7/2 |
| T stage 2 | 29/19 | 37/11 | 17/11 | 20/8 | 12/8 | 17/3 |
| N stage 0 | 35/27 | 43/19 | 24/19 | 29/14 | 11/8 | 14/5 |
| N stage 1 | 14/10 | 21/3 | 6/8 | 11/3 | 8/2 | 10/0 |
| Differentiation | | | | | | |
| Good | 3/1 | 3/1 | 1/1 | 1/1 | 2/0 | 2/0 |
| Moderate | 19/16 | 25/10 | 10/11 | 14/7 | 9/5 | 11/3 |
| Poor | 27/20 | 36/11 | 19/15 | 25/9 | 8/5 | 11/2 |

*Statistically non-significant ($P > 0.05$).

eliminated from survival analysis. There were 73 men and 20 women with a mean age of 60.3 years (s.d. 7.8, range 35–74). Survival analysis was based on 86 patients. By the time this study was undertaken 35 patients had died after a mean (± s.d.) post-operative survival of 459 (± 512) days. Table I shows the characteristics of the 86 patients included in the survival analysis according to the expressional status of MHC class I molecules.

*Tissues*

Representative samples from the tumours were snap frozen in liquid nitrogen and stored at −70°C. Histological diagnosis, evaluation of the differentiation and nodal status were assessed by light microscopy of routinely processed tissue with histochemical and immunohistochemical confirmation when necessary. Classification was performed according to the WHO system (Sobin *et al.*, 1982). Of the 93 cases examined, 61 were squamous carcinomas (SQCs) and 32 adenocarcinomas (ACs). There were four well-differentiated, 39 moderately differentiated and 50 poorly differentiated tumours.

*Monoclonal antibodies*

Immunohistochemical analysis included four monoclonal (W6/32, BBM-1, MA2.1 and HCA2) and a polyclonal (AK1-7) antibody. W6/32 appears to detect an antigenic determinant shared along the HLA-A, B and C loci (Barnstable *et al.*,1978). This determinant is a product of the interaction between the HLA-A, B, C and $\beta_2$-m polypeptide chains (Parham *et al.*, 1979). BBM-1 is a specific monoclonal antibody against $\beta_2$-m (Brodsky *et al.*, 1979). MA2.1 detects HLA-A2 and B17 (McMichael *et al.*, 1980) whereas HCA2 recognises an epitope unique for HLA-A locus heavy chains that is present on the free heavy chain only (Stam *et al.*, 1990). For the detection of TAP-1 protein we used the affinity purified polyclonal antibody AK1-7 raised against the carboxy-terminal peptide of TAP-1 sequence. Two lymphoplasmacytoid cell lines were used as negative and positive controls respectively for the specificity of this antibody. The first is the mutant LCL721.174, which has deleted both the TAP genes, and the second the wild-type LCL721. (Kelly *et al.*, 1992; Spies *et al.*, 1992). This antibody was used at a dilution 1:100. In the mutant cell line there was no immunoreactivity with either the W6/32 or the AK1-7 antibodies implying that in the absence of the transporter molecule there was no functional expression of the MHC class I molecules. The wild-type cell line showed strong cytoplasmic positivity with the AK1-7 antibody and expression of W6/32 was always detected.

*Immunohistochemistry*

Cryostat sections (7 mm thick) were fixed in acetone for 10 min at room temperature, left to dry overnight and stored at −20°C until required for staining. Immunohistochemical staining was performed using the alkaline phosphatase–anti-alkaline phosphatase (APAAP) method as described previously (Cordell *et al.*, 1984). For the polyclonal antibody AK1-7 a single modification of this technique was made, using an incubation step of mouse-anti-rabbit immunoglobulin.

*Assessment of staining*

Microscopic examination of immunohistochemically stained sections was carried out by two observers. The whole section was screened for the distribution of HLA antigens but areas of obvious tumour necrosis were avoided for counting. Normal respiratory epithelium and inflammatory lymphoid cells were used in each case as a control. Thus, a particular antigen was only considered to be lost by the tumour if it was still expressed by the adjacent normal respiratory epithelium and the lymphocytes. The evaluation was semi-quantitative. A tumour was scored as negative (−) if less than 10% of the cells were labelled and as positive (+) if more than 75% of the cells were strongly stained. When the percentage of positive neoplastic cells was between 10% and 75% irrespective of the staining intensity the tumour was recorded as showing reduced expression.

*Statistical analysis*

The association between HLA expression and tumour type, degree of differentiation, T stage and N stage was investigated by the use of frequency tables (Altman, 1991). Survival was measured in days from the date of surgery. Actuarial survival curves were plotted using the Kaplan–Meier method (Kaplan and Meier, 1958). The statistical significance was calculated using the log-rank test (Peto *et al.*, 1977) and the hazard ratio with a 95% confidence interval was calculated as described by Machin and Gardner (1989). The homogeneity of age in the various subgroups was assessed by calculating the *F*-value with one-way analysis of variances (Armitage and Berry, 1987).

### Results

Tables I and II summarise the results of the immunohistochemical expression of HLA class I and TAP-1 in the groups of SQC and AC.

**Table II** Survival of 86 patients with non-small-cell lung carcinoma according to MHC class I and TAP-1 protein expression

| *Results of staining* | *No. of patients* | *5 year survival (%)* | *χ-square* | P-*value* | *Hazard ratio (95% CI)* |
|---|---|---|---|---|---|
| W6/32 positive | 49 | 56.6 | | | |
| | | | 0.0001 | >0.95 | 0.99 (0.5–1.96) |
| W6/32 negative | 37 | 55 | | | |
| W6/32 positive MA2.1 positive | 30 | 57.6 | | | |
| | | | 0.2839 | >0.5 | 0.75 (0.26–2.16) |
| W6/32 positive MA2.1 negative | 13 | 53.9 | | | |
| AK1-7 positive | 64 | 54.7 | | | |
| | | | 0.1773 | >0.5 | 1.18 (0.55–2.5) |
| AK1-7 negative | 22 | 56.3 | | | |

**Figure 1** (**a**) W6/32 expression in a squamous cell carcinoma (bar 100 mm). (**b**) MA2.1 selective loss from the same case (bar 50 mm). (**c**) Loss of W6/32 in an adenocarcinoma (bar 100 mm). (**d**) Expression of TAP-1 in the above case (bar 100 mm). Lymphocytes and stromal cells are positive in all the cases shown above.

### *HLA class I and TAP-1 expression*

In the normal lung class I antigens, TAP-1, HCA2, BBM.1 and MA2.1 were expressed by the endothelial cells, lymphocytes, bronchiolar and alveolar epithelium and alveolar macrophages. As far as the tumours are concerned, 37 out of 93 cases showed loss of the framework antigenic determinant, either partial or complete, evidenced by reduced (five cases) or negative (32 cases) staining with W6/32 antibody. The loss was commoner in SQC (27 out of 61 cases) than in AC (10 out of 32 cases), although no statistically significant difference could be reached. Loss of the framework antibody W6/32 was usually accompanied by loss of $\beta_2$-m (28 out of 37 cases) and loss of A locus (19 out of 37 cases). Selective loss of A locus was detected in 2 out of 24 cases positive with W6/32. Selective loss of A2 allele was seen in 13 out of 43 cases in which A2 was present in the adjacent lung. All 13 cases were positive for W6/32.

TAP-1 protein was lost in 22 cases (17 SQC and 5AC). These cases also showed synchronous loss of $\beta_2$-m and/or heavy chain. A locus-isolated TAP-1 defect was not identified in our series. All cases showing loss of TAP-1 molecule were negative for W6/32 as well. No relationship could be found between the mode of MHC class I antigen and TAP-1 expression on the one hand and histological type, degree of differentiation, tumoral or nodal stage on the other, even when the last three parameters were examined with each histological group separately (Figure 1).

### *Survival analysis*

The results of the survival analysis on the series of 86 non-small-cell lung carcinoma according to the staining with the antibodies W6/32, TAP-1 and MA 2.1 are summarised in Table II and the survival curves are shown in Figure 2a, b and c. Furthermore we examined whether the selective loss of the A2 allele was of any prognostic value: for this purpose we compared the survival of patients with W6/32- and MA2.1- positive tumours with that of patients with W6/2-

**Figure 2** Survival in relation to loss of expression of TAP-1 (**a**), MA2.1 (**b**) and W6/32 (**c**).

positive and MA2.1- reduced or -negative tumours. No difference emerged. The distribution of ages was homogeneous across all subgroups of patients when analysed, independently of the expressional status of MHC class 1 and TAP-1 molecules (Table II). There was no association of staining with any of these antibodies with tumour differentiation, T stage or N stage (data not shown). Survival curves were plotted also for the other two antibodies, BBM1 and HCA2, but no difference could be found (data not shown). No difference could be detected even when the groups of squamous cell and adenocarcinoma were examined separately (Figure 3).

### Discussion

In the present study we detected two types of alterations in the surface expression of MHC class I antigens by the neoplastic cells: total loss of MHC class I molecule and selective losses of HLA-A locus and A2 allele. Total loss of MHC class I molecule as evidenced by negative reaction to W6/32

**Figure 3** Survival in relation to loss of expression W6/32 in (**a**) squamous cell carcinomas and (**b**) adenocarcinomas.

was detected in 38% of our cases, a figure higher than those quoted in previous studies (Redondo *et al.*, 1991*a*, *b*). Selective losses of A locus and A2 allele were identified in 8.3% and 27% respectively. Our findings show that loss of the assembled molecule is not only due to loss of $\beta_2$-m but also to loss of TAP1 molecules and/or heavy chains.

Previous studies of lung cancer have shown no difference between $\beta_2$-m and heavy chain expression (Doyle, 1985; Redondo *et al.*, 1991*b*). However, in our series we have seen such a difference in a small proportion of cases (9.1%). Such uncoordinated expression of $\beta_2$-m and heavy chains has also been observed in colon carcinomas (Momburg *et al.*, 1989). It is also worthy of note that the failure to detect A locus or A2 allele is not necessarily associated with loss of the assembled class I molecule. This is similar to the situation in colon carcinomas (Rees *et al.*, 1988; Kaklamanis *et al.*, 1992).

Interestingly, loss of the transporter protein was always combined with $\beta_2$-m and/or A locus loss and was invariably associated with lack of expression of the assembled class I molecule. This implies that in the absence of the transporter protein the antigenic peptide is not able to join the MHC class I molecule rendering the assembly of the heavy chains and $\beta_2$-m impossible.

The mechanisms by which total or partial losses of HLA antigens occur are not yet well known. Theoretically, they might reflect underlying chromosomal abnormalities (e.g. translocations or deletions) in the short arm of chromosome 6 for the heavy chains and TAP-1 or chromosome 15 for $\beta_2$-m. However, there is no current evidence to support this. In fact, molecular studies in lung carcinomas have failed to demonstrate rearrangements of class I genes in cases with abnormal surface expression of these antigens (Doyle *et al.*, 1985; Redondo *et al.*, 1991*a*). A more plausible mechanism is that of transcriptional down-regulation of MHC class I genes, which could be related to the action of cellular oncogene products, such as c-myc oncoprotein. This has been shown to operate in SLCL tumours and cell lines (Doyle, 1985). However, in NSCLC expression of class I antigens appears to be independent of c-myc expression. Alternatively, it has been hypothesised that a post-transcriptional mechanism may be involved in the differential expression of HLA-A, B, C products in NSCLC, since there is not always a close relation between the surface expression of these antigens and their mRNAs (Redondo *et al.*, 1991*a*). Moreover, γ-interferon-mediated regulation of HLA-gene subsets has been documented (Haken *et al.*, 1989; Schmidt *et al.*, 1990).

The lack of association between MHC class I loss and degree of differentiation, in our study, is at variance with data from the literature relating a loss of these antigens in lung cancer to the degree of differentiation as well as to the presence of aneuploidy in NSCLC and to an increased mitotic rate (Dämmrich *et al.*, 1990; Redondo *et al.*, 1991*b*). Based upon these findings it has been suggested that MHC class I loss is an indication of a more aggressive phenoptype and of a more rapid tumour growth. However, no relationship with the tumoral or nodal stage was found in the studies of Dämmrich *et al.* (1990) and Redondo *et al.* (1991*b*), in concordance with the results of the present study.

Experiments in murine models have shown that the loss of MHC class I antigen expression allows tumour growth and metastasis formation by escape from T-cell-mediated surveillance (Hui *et al.*, 1984; Tanaka *et al.*, 1985; Wallich *et al.*, 1985). Following this line of argument it was tempting to speculate that tumours lacking the above antigens would be prone to pursue a more unfavourable clinical course as compared with those with normal expression. This idea was further strengthened by the association of MHC class I loss with a poorer degree of differentiation as reported for breast (Wintzer *et al.*, 1990), colon (Momburg *et al.*, 1986) and laryngeal (Lopez-Nevot *et al.*, 1989) carcinomas. Clinical studies however have failed to confirm this idea, at least as far as colorectal carcinomas are concerned (Stein *et al.*, 1988; Möller *et al.*, 1991). In the case of breast cancer, however, the question of prognostic relevancy of MHC class I expression is still open (Wintzer *et al.*, 1990; Concha *et al.*, 1991).

This study shows that down-regulation of antigen-presenting and antigen-transporting molecules is a common phenomenon in NSCLC. Specific allelic loss (A2) was also frequently detected and it might be of interest to study the expression of the entire allelic repertoire present on tumour cells. Although no correlation was found with clinicopathological parameters, the understanding of the underlying mechanisms that are responsible for this defective expression, would be of paramount importance.

**Acknowledgements**
We thank Alain Townsend for supplying the AKI-7 antiserum and Felicity Williams for secretarial assistance.

### References

ALTMAN DG. (1991). Comparing groups: categorical data. In *Practical Statistics for Medical Research.* pp. 229–276. Chapman and Hall: London.

ARCE-GOMEZ B, JONES F, BARNSTABLE C, SOLOMON E AND BODMER W. (1978). The genetic control of HLA-A and B antigens in somatic cell hybrids: requirement for b2-microglobulin. *Tissue Antigens*, **11**, 96–112.

ARMITAGE P AND BERRY G. (1987). *Statistical Methods in Medical Research.* Blackwell: Oxford.

BARNSTABLE CJ, BODMER WF, BROWN G, GAFFRE G, MILSTEIN C, WILLIAMS AF AND ZIEGLER A. (1978). Production of monoclonal antibodies to group A erythrocytes, HLA and other human cell surface antigens-new tools for genetic analysis. *Cell*, **14**, 9–20.

BODMER W. (1987). The HLA system: structure and function. *J. Clin. Pathol.*, **40**, 948–958.

BRODSKY F, BODMER W AND PARHAM P. (1979). Characterisation of a monoclonal anti-b2-microglobulin antibody and its use in the genetic and biochemical analysis if major histocompatibility antigens. *Eur. J. Immunol.*, **9**, 536–545.

CONCHA A, CABRERA T, RUIZ-CABELLO F AND GARRIDO F. (1991). Can the HLA phenotype be used as a prognostic factor in breast carcinoma? *Int. J. Cancer*, (suppl.6), 146–154.

CORDELL JL, FALINI B, ERBER WN, GHOSH AK, ABDULAZIZ Z, MCDONALD S, PULFORD KAF AND MASON DY. (1984). Immunoenzymatic labelling of monoclonal antibodies using immune complexes of alkaline phosphatase and monoclonal anti-alkaline phosphatase (APAAP complexes). *J. Histochem. Cytochem.*, **32**, 219–229.

CROMME F, AIREY J, HEEMELS M, PLOEGH H, KEATING P, STERN P, MEIJER C AND WALBOOMERS J. (1994). Loss of transporter protein, encoded by the TAP-1 gene, is highly correlated with loss of HLA expression in cervical carcinomas. *J. Exp. Med.*, **179**, 335–340.

DAAR A, FUGGLE S, FABRE J, TING A AND MORRIS P. (1984). The detailed distribution of HLA-A, B, C antigens in normal human organs. *Transplantation*, **38**, 287–293.

DÄMMRICH J, MÜLLER-HERMELINK HK, MATTNER A, BUCHWALD J AND ZIFFER S. (1990). Histocompatibility antigen expression in pulmonary carcinomas as indication of differentiation and of special subtypes. *Cancer*, **65**, 1942–1954.

DAUSSET J. (1981). The major histocompatibility complex in man. Past, present and future concepts. *Science*, **213**, 1469–1474.

DOYLE A, MARTIN J, FUNA K, GADGAR A, VARNEY D, MARTIN SE, LINNOILA I, CUCITTA F, MULSHINE J, BANN P AND MINNA J. (1985). Markedly decreased expression of class I histocompatibility antigens, protein and mRNA in human small-cell lung cancer. *J. Exp. Med.*, **161**, 1135–1151.

FESTENSTEIN H AND GARRIDO F. (1986). MHC antigens and malignancy (news). *Nature*, **322**, 502–503.

FUNA K, GAZDAR AF, MINNA JD AND LINNOILA I. (1986). Paucity of b2-microglobulin expression on small cell lung cancer, bronchial carcinoids and certain other neuroendocrine tumors. *Lab. Invest.*, **55**, 186–193.

GOEPEL JR, REES RC, ROGERS K, STODDARD CJ, THOMAS WEG AND SHEPHERD L. (1991). Loss of monomorphic and polymorphic HLA antigens in metastatic breast and colon carcinoma. *Br. J. Cancer*, **64**, 880–883.

HAKEN R, LE BOUTELIER P, BARAD M, TRUJILLO M, MERCIER P, WIETZERBIN J AND LEMONIER FA. (1989). IFN-mediated differential regulation of the expression of HLA-B7 and HLA-A3 class I genes. *J. Immunol.*, **142**, 297–305.

HUI K, GROSVELD F AND FESTENSTEIN H. (1984). Rejection of transplantable AKR leukaemic cells following MHC DNA-mediated cell transformation. *Nature*, **311**, 750–752.

KAKLAMANIS L, GATTER KC, HILL AB, MORTENSEN N, HARRIS AL, KRAUSA P, MCMICHAEL A, BODMER JG AND BODMER WT. (1992). Loss of HLA class-1 alleles, heavy chains and b2-microglobulin in colorectal cancer. *Int. J. Cancer*, **51**, 379–385.

KAKLAMANIS L, TOWNSEND A, DOUSSIS-ANAGNOSTOPOULOU I, MORTENSEN N, HARRIS AL AND GATTER KC. (1994). Loss of major histocompatibility complex-encoded transporter associated with antigen presentation (TAP1) in colorectal cancer. *Am. J. Pathol.*, **145**, 505–509.

KAPLAN EL AND MEIER P. (1958). Non parametric estimation from incomplete observations. *J. Am. Stat. Ass.*, **53**, 457–481.

KARRE K, LJUNGREN HG, PONTEC G AND KIESSLING R. (1986). Selective rejection of H-2-deficient lymphoma variants suggests alternative immune defence strategy. *Nature*, **319**, 675–678.

KELLY A, POWIS SH, KERR L-A, MOCKRIDGE I, ELLIOTT T, BASTIN J, UCHANSKA-ZIEGLER B, ZIEGLER A, TROWSDALE J AND TOWNSEND A. (1992). Assembly and function of the two ABC transporter proteins encoded in the human major histocompatibility complex. *Nature*, **355**, 641–644.

KLEIJMEER MJ, KELLY A, GEUZE HJ, SLOT JW, TOWNSEND A AND TROWSDALE J. (1992). Location of MHC-encoded transporters in the endoplasmic reticulum and cis-Golgi. *Nature*, **357**, 342–344.

LOPEZ-NEVOT MA, ESTEBAN F, FERRON A, OLIVIA MR, ROMERO C, HUELIN C, RUIZ-GABELLO F AND GARRIDO F. (1989). HLA-class-1 gene expression on human primary tumours and autologous metastases: demonstration of selective loss of HLA antigens on colorectal gastric and laryngeal carcinomas. *Br. J. Cancer*, **59**, 221–226.

McMichael A, Parham P, Rust N and Brodsky F. (1980). A monoclonal antibody that recognizes an antigenic determinant shared by HLA-A2 and B17. *Hum. Immunol.*, **1**, 121-129.

Machin D and Gardner MJ. (1989). Calculating confidence intervals for survival time analyses. In *Statistics with Confidence: Confidence Intervals and Statistical Guidelines*. pp. 64-70. British Medical Journal: London.

Moller P, Momburg F, Koretz K, Moldenhauer G, Herforth C, Otto HF, Hammerling G and Schlag P. (1991). Influence of major histocompatibility complex class-I and II antigens on survival colorectal carcinoma. *Cancer Res.*, **51**, 729-736.

Momburg F, Degener T, Bacchus F, Moldenhauer G, Hämmerling GJ and Möller P. (1986). Loss of HLA-A, B, C and *de novo* expression of HLA-D in colorectal carcinoma. *Int. J. Cancer*, **38**, 459-464.

Momburg F, Ziegler A, Harpprecht J, Möller A, Moldenhauer G and Hammerling G. (1989). Selective loss of HLA-A or HLA-B antigen expression in colon carcinoma. *J. Immunol.*, **142**, 352-358.

Orgad S, Yang SY, Gazit E, Relias V, Zaizov R, Lyson S and Yunis EJ. (1985). Expression of extra class I major histocompatibility antigens on T-cell acute lymphoblastic leukemia (ALL) lymphoblasts. *Hum. Immunol.*, **12**, 133-141.

Ortman B, Androlewicz MJ and Cresswell P. (1994). MHC class I/b2-microglobulin complexes associate with TAP transporters before peptide binding. *Nature*, **368**, 864-867.

Parham P, Barnstable CJ and Bodmer WF. (1979). Use of a monoclonal antibody (W6/32) in structural studies of HLA-A, B, C antigens. *J. Immunol.*, **123**, 342-349.

Peto R, Pike MC, Armitage P, Breslow NF, Cox DR, Howard SV, Mantel N, McPherson K, Peto J and Smith PG. (1977). Design and analysis of randomised clinical trials requiring prolonged observation of each patient. *Br. J. Cancer*, **35**, 1-39.

Ploegh H, Orr H and Strominger. Major histocompatibility antigens: the human (HLA-A, B, C) and murine (H-2K, H-2D) class-1 molecules. *Cell*, **24**, 287-299.

Redondo M, Ruiz-Cabello F, Concha A, Cabrera T, Perez-Ayala M, Oliva MR and Garrido F. (1991*a*). Altered HLA class I expression in non-small cell lung cancer is independent of c-myc activation. *Cancer Res.*, **51**, 2463-2468.

Redondo M, Concha A, Oldiviela R, Cueto A, Gonzalez A, Garrido F and Ruiz-Cabello F. (1991*b*). Expression of HLA class I and II antigens in bronchogenic carcinomas: its relationship to cellular DNA content and clinical-pathological parameters. *Cancer Res.*, **51**, 4948-4954.

Rees R, Buckle A, Geltsthorpe K, James V, Potter C, Rogers K and Jacob G. (1988). Loss of polymorphic A and B locus HLA antigens in colon carcinoma. *Br. J. Cancer*, **57**, 374-377.

Schmidt H, Gekeler V, Haas H, Engler-Blum G, Steiert I, Probst H and Muller CA. (1990). Differential regulation of HLA class I genes by interferon. *Immunogenetics*, **31**, 245-252.

Sobin LH. (1982). The World Health Organization's histological classification of lung tumors. A comparison of the first and second editions. *Cancer Detect. Prev.*, **5**, 391-406.

Spies T, Cerundolo V, Colonna M, Cresswell P, Townsend A and Demars R. (1992). Presentation of viral antigen by MHC class I molecules is dependent on a putative peptide transporter heterodimer. *Nature*, **355**, 644-646.

Stam NJ, Vroom T, Peters P, Patsoors E and Ploegh H. (1990). HLA-A and HLA-B specific monoclonal antibodies reactive with free heavy chains in Western blots, in formalin-fixed paraffin-embedded tissue sections and in cryo-immuno-electron microscopy. *Int. Immunol.*, **2**, 113-125.

Stein B, Momburg F, Schwaz V, Schlag P, Moldenhauer G and Möller P. (1988). Reduction or loss of HLA-A, B, C antigens in colorectal carcinoma appears not to influence survival. *Br. J. Cancer*, **57**, j364-368.

Tanaka K, Isselbacher K, Khoury G and Jay G. (1985). Reversal of oncogenesis by the expression of a major histocompatibility complex class I gene. *Science*, **228**, 26-30.

Townsend A, Elliot T, Cerundolo V, Foster L, Barber B and Tse A. (1990). Assembly of MHC class I molecules analysed in vitro. *Cell*, **62**, 285-295.

Trowsdale J, Hansen I, Mockridge I, Beck S, Townsend A and Kelly A. (1990). Sequences encoded in the class I region of the MHC related to the 'ABC' superfamily of transporters. *Nature*, **348**, 741-743.

Wallich R, Bulbuc O, Hämmerling G, Katzar S, Segal S and Feldman M. (1985). *De novo* expression of H2-K antigens on metastatic tumour cells following H-2 gene transfection results in abrogation of their metastatic properties. *Nature*, **315**, 301-305.

Wintzer H-O, Benzing M and von Kleist S. (1990). Lacking prognostic significance of b2-microglobulin, MHC class land class II antigen expression in breast carcinomas. *Br. J. Cancer*, **62**, 289-295.

Zinkernagel RM and Doherty P. (1979). MHC-restricted cytotoxic T cells: studies on the biological role of the polymorphic major transplantation antigens determining T-cell restriction-specificity, function and responsiveness. *Adv. Immunol.*, **27**, 51-177.